# The Role of Multimodality Imaging in Pediatric Cardiomyopathies

**DOI:** 10.3390/jcm12144866

**Published:** 2023-07-24

**Authors:** Sara Moscatelli, Isabella Leo, Francesco Bianco, Nunzia Borrelli, Matteo Beltrami, Manuel Garofalo, Elena Giulia Milano, Giandomenico Bisaccia, Ferdinando Iellamo, Pier Paolo Bassareo, Akshyaya Pradhan, Andrea Cimini, Marco Alfonso Perrone

**Affiliations:** 1 Inherited Cardiovascular Diseases, Great Ormond Street Hospital for Children NHS Foundation Trust, London WC1N 3JH, UK; sara.moscatelli@gosh.nhs.uk; 2 Paediatric Cardiology Department, Royal Brompton and Harefield Hospitals, Guy’s and St. Thomas’ NHS Foundation Trust, London SW3 5NP, UK; 3 Department of Experimental and Clinical Medicine, Magna Graecia University, 88100 Catanzaro, Italy; i.leo@rbht.nhs.uk; 4 Cardiology Department, CMR Unit, Royal Brompton and Harefield Hospitals, Guys’ and St. Thomas’ NHS Trust, London SW3 5NP, UK; 5 Cardiovascular Sciences Department—AOU “Ospedali Riuniti”, 60126 Ancona, Italy; francesco.bianco@ospedaliriuniti.marche.it; 6 Adult Congenital Heart Disease Unit, A.O. dei Colli, Monaldi Hospital, 80131 Naples, Italy; nunziaborrelli16@gmail.com; 7 San Giovanni di Dio Hospital, 50143 Florence, Italy; beltrami.matteo1@gmail.com; 8 Department of Clinical and Experimental Medicine, Careggi University Hospital, 50134 Florence, Italy; maanu.507@gmail.com; 9 Centre for Cardiovascular Imaging, Great Ormond Street Hospital for Children NHS Foundation Trust, London WC1N 3JH, UK; elena.milano@gosh.nhs.uk; 10 Department of Neuroscience, Imaging and Clinical Sciences, “G.d’Annunzio” University of Chieti-Pescara, 66100 Chieti, Italy; bisacciagiandomenico@gmail.com; 11 Division of Cardiology and Cardio Lab, Department of Clinical Sciences and Translational Medicine, University of Rome Tor Vergata, 00133 Rome, Italy; iellamo@uniroma2.it; 12 School of Medicine, University College of Dublin, Mater Misericordiae University Hospital and Children’s Health Ireland Crumlin, D07 R2WY Dublin, Ireland; piercard@inwind.it; 13 Department of Cardiology, King George’s Medical University, Lucknow 226003, India; akshyaya33@gmail.com; 14 Nuclear Medicine Unit, St. Salvatore Hospital, 67100 L’Aquila, Italy; andreacimini86@yahoo.it; 15 Clinical Pathways and Epidemiology Unit, Bambino Gesù Children’s Hospital IRCCS, 00165 Rome, Italy

**Keywords:** pediatric cardiomyopathy, cardiovascular multimodality imaging, pediatric cardiology

## Abstract

Cardiomyopathies are a heterogeneous group of myocardial diseases representing the first cause of heart transplantation in children. Diagnosing and classifying the different phenotypes can be challenging, particularly in this age group, where cardiomyopathies are often overlooked until the onset of severe symptoms. Cardiovascular imaging is crucial in the diagnostic pathway, from screening to classification and follow-up assessment. Several imaging modalities have been proven to be helpful in this field, with echocardiography undoubtedly representing the first imaging approach due to its low cost, lack of radiation, and wide availability. However, particularly in this clinical context, echocardiography may not be able to differentiate from cardiomyopathies with similar phenotypes and is often complemented with cardiovascular magnetic resonance. The latter allows a radiation-free differentiation between different phenotypes with unique myocardial tissue characterization, thus identifying the presence and extent of myocardial fibrosis. Nuclear imaging and computed tomography have a complementary role, although they are less used in daily clinical practice due to the concern related to the use of radiation in pediatric patients. However, these modalities may have some advantages in evaluating children with cardiomyopathies. This paper aims to review the strengths and limitations of each imaging modality in evaluating pediatric patients with suspected or known cardiomyopathies.

## 1. Introduction

Cardiomyopathies are rare in children (age < 18 y/o), with an estimated incidence of about 1 per 100,000 person-years [1]. However, the estimated incidence is higher when considering children diagnosed before one year of age (8 cases per 100,000 person-years), with male children and children of Black ethnicity more frequently affected [2]. Despite being rare, the prognosis of this condition is poor, with approximately 40% of children undergoing transplantation or dying within two years from the diagnosis [1]. Despite the advances in the diagnostic approach and in medical therapies, outcomes have not significantly improved in the last decades, with cardiomyopathies still representing the leading cause of heart transplantation in children [3,4,5]. According to large international registries, the most frequent phenotype is dilated cardiomyopathy (DCM, 50% of cases), followed by hypertrophic cardiomyopathy (HCM, 35–50% of cases), whilst restrictive phenotype, left ventricular non-compaction, and mixed phenotypes are much rarer [5]. Since the first classification, published in 1980 by the World Heart Organization [6], several revised versions have been provided over the years, with no current consensus between European and American Guidelines [7,8]. However, a recent scientific statement by the American Heart Association suggests a hierarchy-based classification that starts from the morpho-functional characteristics and further subcategorizes the disease accordingly to the presence or absence of identifiable genetic causes [5]. Cardiac imaging is therefore key in evaluating cardiac phenotypes, allowing classification, in screening assessments for carriers of genetic abnormalities, in risk stratification, and response to treatment. Echocardiography is often the first-line imaging modality used for this purpose. Cardiac magnetic resonance (CMR) has, however, an additional and unique role, allowing not only a functional assessment but also myocardial tissue characterization. This review aims to provide an overview of the strengths and limitations of different imaging modalities with evidence about their use in children affected by cardiomyopathies.

## 2. Transthoracic Echocardiography in Cardiomyopathy

The non-invasive nature, high spatiotemporal resolution, and wide availability of transthoracic echocardiography (TTE) make this technique an effective imaging tool for the diagnosis and follow-up of patients with known or suspected cardiomyopathy. The appropriate frequency of follow-up imaging in cardiomyopathies is patient-specific and TTE screening for children and adolescents from genotype-positive families should be performed every 1–2 years.

A variety of traditional and advanced methods is used for the contemporary TTE evaluation of patients with cardiomyopathies. While two-dimensional (2D) and three-dimensional (3D) TTE allow the estimation of chamber dimension and ventricular function, colour, tissue, pulse, and continuous wave Doppler allow for the evaluation of valvular function. Additionally, torsion echocardiography and speckle tracking provide the assessment of ventricular mechanics and synchronization [9].

### 2.1. Protocols

#### 2.1.1. Two-Dimensional Echocardiography

The quantification of left ventricle (LV) size and systolic function by 2D TTE provides useful diagnostic, prognostic, and therapeutic information. Either a parasternal long-axis view or a short-axis view can be used to obtain the 2D mode or M-mode end-diastolic and end-systolic LV size and thickness. In the pediatric population, the LV dimensions, thicknesses, and volumes are reported as z-scores in relation to patient age and body size. The LV ejection fraction (EF) is calculated by measuring the LV volume. The Simpson biplanar method, with the manual recording of the endocardial borders from the apical 4-chamber and apical 2-chamber views, is the suggested technique for this measurement [10,11,12]. Although echocardiographic LV volumes tend to be underestimated due to geometric assumptions and image quality limitations, 2D TTE-derived LVEF correlates well with CMR-derived LVEF [13].

#### 2.1.2. Three-Dimensional Echocardiography

Because 3D TTE volume evaluation has less temporal variability and does not rely on geometric assumptions, it demonstrates a greater correlation with CMR than 2D evaluations. In patients with cardiomyopathies, LV volume and mass quantification by 3D TTE is reliable and reproducible. By avoiding apical foreshortening, 3D TTE has demonstrated greater accuracy in LVEF measurements than 2D TTE, although there is still a small volume underestimation when compared to CMR imaging [14]. Moreover, 3D TTE EF and volumes carry stronger associations with all-cause mortality and cardiac hospitalization than those evaluated by 2D TTE imaging [15]. The 3D TTE evaluation of the left atrium revealed good accuracy, with a high correlation with CMR imaging and prognostic value for the identification of significant adverse cardiac events and atrial tachyarrhythmias [16,17]. Recently, good reproducibility has also been demonstrated for the evaluation of the right ventricle volume by 3D TTE, albeit with significant underestimation compared to CMR [18].

#### 2.1.3. Spectral and Tissue Doppler Imaging

Transmitral filling patterns and diastolic performance have functional and prognostic implications in cardiomyopathies and serial tests allow the monitoring of the decline in function over time [9]. The standard Doppler assessment includes transmitral inflow velocities, the septal and lateral early diastolic velocity of the mitral annulus by tissue doppler imaging (TDI), and pulmonary vein velocities. These measurements, in addition to the evaluation of the left atrium volume index and peak tricuspid regurgitation velocity, allow for the LV diastolic function assessment. In particular, the E/e’ ratio can identify LV relaxation dysfunction in patients with cardiomyopathies and guide treatment [19,20], although in the pediatric population the exact threshold is not well-defined.

TDI is also used in the evaluation of systolic function. The peak systolic annular velocity (S′ wave) allows for a longitudinal systolic function assessment and the early diagnosis of hypertrophic cardiomyopathies in genotype-positive patients [21,22]. Myocardial performance index (MPI) is a time interval index derived from pulsed Doppler. It is defined as the sum of isovolumetric contraction time and isovolumetric relaxation time and provides an estimate of global systolic and diastolic function independent of LV geometry and from the heart rate. Normal values in children aged 3 to 18 years were 0.33 ± 0.02.

#### 2.1.4. Speckle Tracking Echocardiography

Speckle tracking echocardiography (STE) analyzes cardiac deformation by tracking the movement of speckles within the myocardium. It has been shown to be a sensitive tool for the early detection of myocardial dysfunction and can provide valuable information for the diagnosis, prognosis, and management of cardiomyopathy [23,24].

In addition, the study of left atrial strain (LAS) has been used to analyze diastolic function in children with cardiomyopathies [25], being also able to predict atrial fibrillation and heart failure [26,27].

STE can be used to assess myocardial torsion by analyzing the deformation of the myocardium in multiple planes. Myocardial torsion refers to the rotation of the myocardium around its long axis during the cardiac cycle. It has been studied in various types of cardiomyopathies as a measure of myocardial function [28,29]. Of note, one of the limitations of STE is its dependency on loading conditions. More recently a novel index of myocardial performance implementing hemodynamic parameters to strain analysis has been proposed to estimate non-invasively myocardial work parameters. Despite the fact that large studies on its usefulness in children with cardiomyopathies are currently lacking, these indices have demonstrated feasibility and reproducibility in the pediatric population, providing additional information in several clinical settings [30,31].

### 2.2. Echocardiography Application to Paediatric Cardiomyopathies

**Figure 1:**
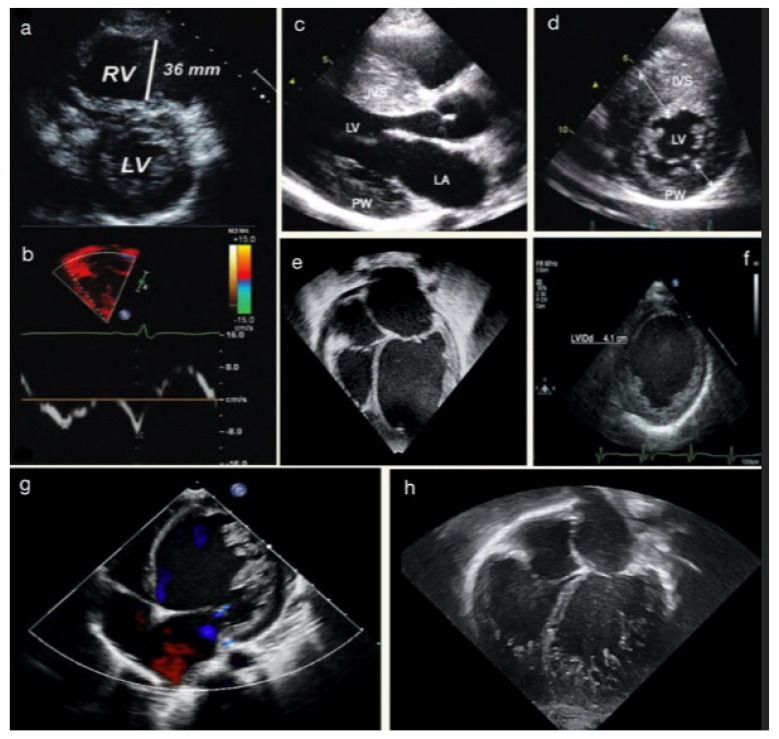
(**a**) Parasternal short-axis view in a 14-year-old female with ventricular tachycardia during exercise, demonstrating right ventricular (RV) enlargement with suspected ACM. (**b**) TDI of the RV free-wall showing diminished tricuspid annular TD velocities of the same patient. (**c**) Parasternal long-axis view in a 13-year-old male with SIV hypertrophy in HCM. (**d**) Parasternal short-axis view showing hypertrophy of mid-ventricular IVS. (**e**) Apical-4-chamber view showing dilated LV and LA in suspected DCM. (**f**) Parasternal short-axis view demonstrates LVEDD at the papillary muscles level. The images (**g**,**h**) show two different cases of LVNC.

TTE allows for assessing the main feature of HCM, from the severity and pattern of LV hypertrophy (LVH) to the possible obstruction in the LV outflow tract (LVOTO) and mitral valve abnormalities (Figure 1). The first indicator of HCM is the existence of a localized or generalized thickness of ventricle walls in the absence of other conditions that might result in pressure overload or an infiltrative state. Even though, in children with HCM specific z-scores thresholds have not been independently verified, those who are asymptomatic and have no family history may need a z-score > 2.5 to be diagnosed with early HCM, and those who are genotype-positive or have a clear family history may only need a z-score > 2 [32,33]. The disease is frequently accompanied by several additional anatomical and functional abnormalities, such as myocardial crypts, LVOT obstruction, and mitral valvular abnormalities. The latter include systolic anterior movement of the anterior leaflet, leaflet elongation, abnormal septal chordal insertions, and the anterior displacement of the papillary muscle. LVOT obstruction may not be present at rest, however, stress echocardiography may help to reveal this obstruction and stratify children at higher risk [34].

Ventricular systolic function is generally normal or hyperkinetic. LVEF < 50% indicates severe LV systolic dysfunction and correlates with higher rates of adverse events, including all-cause mortality and cardiac transplantation [35,36]. During Doppler examination, HCM patients show longer LV isovolumetric contraction and ejection time, delayed peak systolic velocity, and a reduced stroke volume index due to a smaller LV cavity. GLS has been used as an early marker of systolic dysfunction in genotype-positive phenotype-negative patients. It carries incremental prognostic value, being associated with adverse outcomes and progression to heart failure [37,38]. Moreover, pediatric studies on myocardial torsion have demonstrated an abnormal basal rotation with preserved or increased apical rotation [39]. Diastolic dysfunction is a common finding in symptomatic patients with HCM, and it results from increased myocardial stiffness, impaired LV relaxation, and left atrial dysfunction. Although relaxation anomalies are frequent in children, restrictive physiology is often absent and left atrial dilation is far less prevalent than in adults [5,40]. In addition, left atrial dysfunction, measured by LAS, may have prognostic implications in children with HCM; indeed, it was found to correlate with poor exercise capacity [41].

The two main characteristics of dilated cardiomyopathy (DCM)—cardiac enlargement and decreased systolic function—can both be detected by echocardiography (Figure 1). In children, measure-adjusting for body size is required; hence, the diagnosis of cardiac dilatation is based on LV end-diastolic diameter (LVEDD) and LV end-systolic diameter (LVESD) z-scores > 2. Additional morphological characteristics of DCM include increased sphericity and lower wall thickness-to-cavity dimension ratios of the LV brought on by a relative or real thinning of the ventricular walls. Mitral regurgitation may be present as a result of an enlarged cardiac cavity and dilated mitral annulus [5,40]. In children with DCM, LVEDD has been shown to independently correlate with disease progression and increased risk of heart transplant [42]. Regarding STE analysis, children with DCM present reduced values of longitudinal, radial, and circumferential strain; ventricular twist is reduced as well, due to a lower value of apical rotation [37,43]. Diastolic dysfunction is generally present, with an increased value of the E/E’ ratio [44]. Moreover, MPI z-score ≥ 2 has been associated with earlier poor outcomes [45].

Echocardiography is generally the first approach to studying left ventricular non-compaction cardiomyopathy (LVNC). The hallmark of the disease is the presence of LV trabeculations and deep intratrabecular recesses, in the absence of any coexisting cardiac anomalies (Figure 1). The TTE criteria of LVNC include [46]:

- Jenni criteria: the ratio of noncompacted to compacted myocardium in systole;
- Chin criteria: epicardial surface to trabeculation trough divided by epicardial surface to trabeculation peak in end-diastole;
- Stöllberger criteria: number of trabeculations that move synchronously with myocardium in end-diastole.

The efficacy of STE as a follow-up and screening tool is substantial due to its ability to detect changes in myocardial mechanics as early as childhood and continuing into adulthood. In different pediatric studies, longitudinal, circumferential, and radial strain were found to be reduced and ventricle twist was decreased as a result of lower apical rotation [37,43].

Arrhythmogenic cardiomyopathy (ACM) is characterized by myocardial fibro-fatty replacement leading to heart failure and arrhythmias (Figure 1). The disease was initially called arrhythmogenic right ventricular dysplasia, however, the 2020 international classification of ACM (the “Padua criteria”) recognized three phenotypic variants: dominant-right, biventricular, and dominant-left variant, depending on the ventricle mainly involved [46]. Recently, “Padua criteria” were proven to be accurate even in the pediatric setting [47]. In young patients with arrhythmogenic cardiomyopathy, right ventricle S’ wave and longitudinal strain, both global and free-wall, have been found to be reduced [37,43,48,49].

### 2.3. Limitations and Pitfalls

Echocardiography is still an operator-dependent imaging modality, particularly in the context of pediatric assessment due to the scarcity of dimensional references and normal thresholds, along with the requirement for z-score values. This is particularly crucial to consider in patients with poor acoustic windows and in borderline conditions, like HCM-mimicking condition (hypertrophy in infants from diabetic mothers or in chronic kidney disease, especially dialysis-dependent), or in apical hypertrophy, where the diagnosis can be missed due to difficulties in visualizing the apex. Furthermore, the loading situation and intraobserver/interobserver variability may have an impact on the accuracy of the ventricular function assessment by standard 2D TTE. The evaluation of the right ventricle is technically demanding due to the anterior retrosternal position, peculiar geometry, and pronounced trabeculation. TDI presents specific limitations, including the angle of insonation and the impact of loading circumstances. The advanced echocardiographic techniques (STE, 3D echocardiography) look promising, albeit requiring high-quality images.

## 3. Cardiovascular Magnetic Resonance (CMR) in Pediatric Cardiomyopathies

CMR is an advanced cardiovascular imaging technique that can evaluate cardiac anatomy, function, and hemodynamics without using ionizing radiation. In the context of pediatric cardiomyopathy, CMR is particularly valuable in (1) LV and RV volumetric and functional analysis and in (2) myocardial tissue characterization [49,50]. A CMR study consists of the acquisition of multiple image sequences, which can investigate the different myocardial tissue characteristics, such as the presence of oedema, fibrotic/scarring, or abnormal iron or fat myocardial storage. Although myocardial biopsy remains the gold standard for the pathological assessment of the myocardium, the use of CMR tissue characterization techniques allows a non-invasive assessment of the myocardium and can support the diagnosis in many clinical scenarios. Furthermore, characterizing the myocardium is crucial for assessing the risk of adverse cardiac events and predicting patients’ prognosis, leading to significant changes in clinical management [51,52]. CMR can be performed in any age group; however, depending on the clinical question, when a patient cooperation is needed for image acquisition (breath holding), general anesthesia might be necessary in order to acquire full diagnostic data. This requires a highly specialized anesthetic and clinical team. In large-volume pediatric centers, patients tend to have their first CMR around the age of 8–9, when they can undergo the exam without anesthesia. CMR is typically repeated if new clinical symptoms arise or before the patient is referred to an adult service.

### 3.1. CMR Sequences for Paediatric Cardiomyopathies

To evaluate pediatric cardiomyopathies, the CMR protocol is tailored depending on the clinical question and on the degree of patient cooperation, and it is usually performed as follows:

- Initial localizers images;
- Functional and volumetric assessment with long-axis and short-axis cine (balanced steady-state free precession—bSSFP);
- Pre-contrast oedema sequence (if required);
- Pre-contrast parametric mapping (if required);
- Contrast administration;
- Post-contrast early gadolinium enhancement (if required);
- Post-contrast late gadolinium enhancement.

#### 3.1.1. Initial Localizers

The CMR protocol typically begins with dark- and bright-blood single-shot images. Dark- and bright-blood single-shot images are taken in the axial, coronal, and sagittal planes of the thorax, providing a foundation for planning further sequences and collecting information about structures outside the heart. Depending on the child’s cooperation, these images can be obtained during a breath-hold or free-breathing [53,54]. These initial images are also extremely useful to screen for extracardiac findings.

#### 3.1.2. Cine (bSSFP)

Cine images are typically acquired during a breath-hold, preferably at end-expiration, and are the sequences dedicated to the assessment of LV and RV volumes, function and regional wall motion abnormalities. They are usually acquired in standardized planes similar to the standard echo views including four-chamber, LV three chambers, LV two chambers short-axis (SAX), RV two chambers long-axis, and RV outflow tract. In addition, cine images can be planned on any desirable plane depending on the finding and clinical questions.

These images permit the assessment of wall thickness as well as regional and global systolic function and are also used to calculate both the LV and RV end-diastolic and end-systolic volumes and determine the ejection fraction [53,54,55].

Cine image acquisition can be challenging to obtain in young children as breath holding is required. End-inspiration acquisition could be tried instead, as it is better tolerated than the end-expiration used in adults. Novel free-breathing cine sequences are another alternative option which is becoming increasingly available.

The specific sequences for analyzing tissue characteristics can be performed based on the initial suspicion that led to pediatric patients undergoing a CMR. The most important sequences are listed below.

#### 3.1.3. T2 Weighted Images (Oedema Assessment)

The assessment of myocardial oedema can be obtained using CMR sequences that visualize the water content of the myocardial extracellular space. The intrinsic contrast seen in these images is based on the relaxation properties of protons following radiofrequency pulses; water-bound protons have a lengthy T2 relaxation time, which creates a water-specific contrast when T2-weighted sequences are applied, resulting in a high signal intensity of edematous tissue. The most commonly used sequences to visualize oedema are short-tau triple-inversion recovery prepared fast spin echo sequences (STIR), which enhance the contrast generated by the presence of oedema whilst also suppressing the signal from fat and blood [56,57].

By using oedema sequences, we can assess the presence and extent of myocardial oedema. The distribution pattern of oedema tends to correlates with its etiology, similarly to late gadolinium enhancement (LGE) imaging. Global, patchy, or subepicardial regional distribution patterns typically arise from non-ischemic injury, while ischemic injuries typically show a transmural or subendocardial distribution and follow a coronary distribution. These sequences require breath-holding and are thus challenging in small children.

Oedema images should be acquired in the same imaging planes as the cine images to facilitate a comparison of the functional and anatomical abnormalities present [58,59,60,61].

#### 3.1.4. Native T1,T2 Mapping

Parametric mapping can provide additional myocardial tissue characterization. Indeed, different pathophysiological processes can result in different types of expansion of the extracellular space, whether this is water, fat, iron or fibrosis. This technique is based on the different relaxation times of different myocardial components and involves creating a parametric map from a series of co-registered images [62,63,64,65]. Native T1 mapping and T2 mapping are non-contrast sequences [65].

The main limitation of parametric mapping in children is related to the heart rate (typically higher in children than adults), which may create technical challenges in the acquisition of the images, in addition to the need for breath-holding. Also, children typically have thinner myocardial walls compared to adults, which can create challenges in image analysis and interpretation [58,63,64]. The values obtained from T1 and T2 mapping vary based on the magnetic field strength (1.5 and 3 T), acquisition sequence, and variation between scanners. As such, it is recommended to use local reference ranges obtained from healthy volunteers [62].

#### 3.1.5. Late Gadolinium Enhancement (LGE)

A T1 weighted gradient echo inversion recovery is typically used to perform LGE imaging about 10–20 min after the injection of gadolinium-chelate contrast agent (GBCA). The images acquired early (<3 min) after contrast injection are useful to rule out/in the presence of intracavity thrombus [54] as both the myocardium and cavity will appear enhanced as a result of the contrast injection, whilst the thrombus will appear hypoenhanced as it is avascular.

The different wash-in and washout contrast kinetics in normal and abnormal myocardium result in myocardial enhancement, which is the result of the accumulation of the contrast agent in the extra-cellular space. The distribution pattern of the contrast agent can provide helpful information for identifying different types of cardiomyopathies [58]. For example, ischemic cardiomyopathy is in keeping with subendocardial or transmural late enhancement, while septal mid-wall late enhancement is described in dilated cardiomyopathy. In hypertrophic cardiomyopathy, the pattern of late enhancement tends to be patchy mid-wall within the hypertrophied segments. In myocarditis, the pattern of LGE can be epicardial, typically involving the inferolateral walls or mid-wall [66,67].

LGE assessment is also essential in the workup of patients with suspected or known arrhythmogenic cardiomyopathy. According to the new diagnostic Padua Criteria for arrhythmogenic cardiomyopathy (ACM), transmural LGE is observed in the RV, while in the LV a mid-wall/epicardial stria (ring-like) pattern can be seen [46].

LGE sequences can be acquired in breath-hold or free breathing and ideally in the same planes as the cine and oedema images for direct comparison.

### 3.2. CMR Application in Paediatric Cardiomyopathies

**Figure 2:**
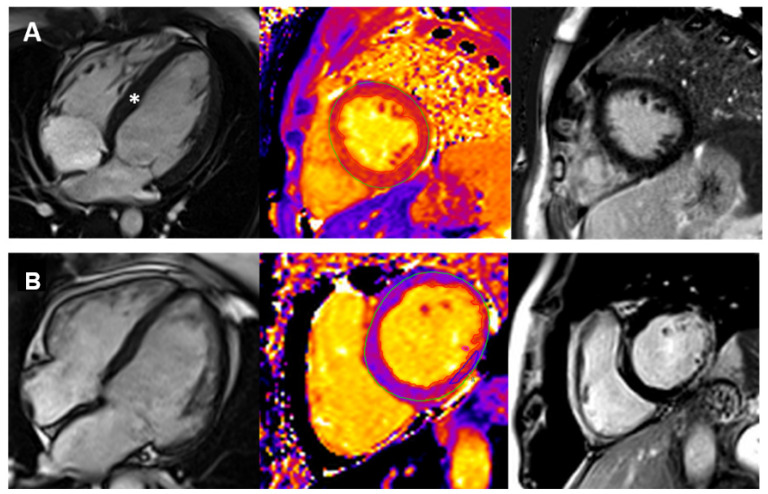
Cardiovascular Magnetic Resonance Imaging in three patients with different cardiomyopathy phenotypes. Panel (**A**): early hypertrophic cardiomyopathy (HCM). There is concentric left ventricular hypertrophy (**left**), accompanied by prolonged T1 mapping values (**center**) and no late gadolinium enhancement. The white asterisk (**left**) indicates the hypertrophic septum. Panel (**B**): dilated cardiomyopathy (DCM) in a patient with Duchenne muscular dystrophy. There is moderate chamber enlargement and LV systolic dysfunction (LVEF 37%) (**left**). T1 mapping values are globally elevated (**center**), and there is mainly epicardial late enhancement of the basal to mid lateral wall (**right**).

CMR imaging is increasingly used in pediatric patients with suspected HCM. CMR can provide an accurate assessment of maximal wall thickness, LVH distribution, and systolic function, enabling differentiation between HCM and its phenocopies based on tissue characterization (Figure 2). Furthermore, CMR complements echocardiography in evaluating the presence of resting left ventricular outflow tract (LVOT) obstruction, mitral systolic anterior motion, if any [39], and quantifies the degree of mitral regurgitation.

Assessing LVH and wall thickness poses challenges in children as there is not a cutoff value distinguishing normal vs. increased thickness. The EF in children with HCM is typically normal or dynamic, accompanied by reduced global longitudinal strain [39].

There is a paucity of evidence regarding the use of CMR to interrogate diastolic dysfunction in children with HCM, and it is unclear whether CMR can provide incremental information with respect to echocardiography in this context [39].

The range of CMR tissue characterization abnormalities observed in children with HCM ranges from high native T1 mapping values to a prevalence of myocardial fibrosis, as high as 46% among patients with overt disease. Despite significant variability, fibrosis typically has a patchy mid-wall pattern involving the hypertrophic segments and/or the RV/LV insertion points. The presence of LGE has been linked to an increased risk of hard cardiac events among pediatric patients [68,69,70].

Among HCM phenocopies, those to be considered in children include mitochondrial cardiomyopathies, Pompe disease, and other glycogen storage diseases, PRKAG2 cardiomyopathy, and lysosomal diseases, including Danon cardiomyopathy [71]. CMR can effectively distinguish HCM from LVH due to Danon disease, a rare X-linked multisystem, extensive LGE pattern sparing the septum [72].

Despite the potential prognostic role of CMR in pediatric HCM, there are no validated risk models currently available that include CMR parameters, and further studies are warranted.

CMR is a valuable tool in assessing dilated cardiomyopathy (DCM) in the pediatric population, providing information on myocardial wall thinning, diffuse trabeculations, global hypokinesia, and quantifying the ejection fraction. By utilizing tissue characterization with LGE, CMR can detect the presence of a linear mid-wall LGE in advanced forms of DCM, usually in the interventricular septum (“mid-wall fibrosis”), which is reported in approximately 30% of DCM cases of all ages and is associated with poor prognosis [4,73,74,75].

CMR is also used to assess familial, genetic, or mitochondrial DCM [75]. Furthermore, it is utilized to assess the extent of myocardial fibrosis and cardiac function in neuromuscular disorders (Figure 2), aiding in identifying those with Duchenne muscular dystrophy who are at risk of developing progressive heart failure [51,74]. However, the need for follow-up scans and primary prevention of sudden cardiac death (SCD) in pediatric patients with DCM is unknown, and future studies should investigate these aspects [51,74].

In assessing pediatric cases of ACM, CMR can detect the morpho-functional data currently part of the Padua Criteria for the diagnosis of ACM. These include: RV akinesia, dyskinesia, or bulging, associated with either RV dilatation or dysfunction; LV systolic dysfunction indicated by depression of LV ejection fraction or reduction in LV global longitudinal strain, with or without LV dilatation; regional LV wall motion abnormalities, such as hypokinesia or akinesia (rarely dyskinesia), with preserved LV systolic function; regional RV LGE; and non-ischemic LV myocardial LGE/fibrosis [46,76]. Myocardial inflammation is another possible finding in pediatric ACM, characterizing the so-called hot phases, thus requiring tailored edema and inflammation imaging, including STIR, T1, and T2 mapping [77,78].

LVNC is a rare phenotype in neonates and children, with limited evidence regarding its etiology, clinical features, and prognostic significance. CMR imaging criteria have been proposed to identify LVNC cases, such as the Petersen criteria, which utilize a ratio of non-compacted to compacted myocardium with a threshold of 2.3 [79] but these have limitations. Although the presence and extent of LV trabeculations have not been demonstrated to impact prognosis in cases of LV dysfunction, follow-up imaging with CMR is recommended due to its higher spatial resolution and increased measurement accuracy compared to echocardiography [79].

### 3.3. Limitations and Pitfalls

There are relative and absolute contraindications of CMR. Typically, ferromagnetic implants are contraindicated (cerebral metallic clip) and non-MRI conditional devices like pacemakers or ICDs.

However, recent data suggest that MRI can be performed on both MR conditional and non-conditional devices in centers with high expertise. GBCA is a crucial component in the characterization of myocardial sequences [80,81]. As with other non-gadolinium-based contrast media, their administration of contrast carries a small risk of allergic reactions, though rarely severe (0.07% in total) [82]. The administration of GBCA in patients with severe renal impairment (eGFR < 30 mL/min/1.73 m^2^) is contraindicated for the small risk of developing nephrogenic systemic fibrosis (NSF), a rare but severe condition. Multiple administrations of GBCA in short time frames have also been linked to the accumulation of gadolinium in the brain’s basal ganglia [83], however, the clinical relevance of these findings has yet to be determined.

Acquiring a full CMR scan requires a high level of patient compliance, which is not always achievable in the pediatric population, however, thanks to the free breathing sequence, and in the presence of a highly experienced team, the CMR protocol can be tailored or shortened and diagnostic data can be acquired in most cases without the need for general anesthesia.

## 4. Computed Tomography in Pediatric Cardiomyopathies

Computed tomography (CT), and in particular cardiovascular computed tomography (CCT), has been progressively used in the evaluation of cardiomyopathies, principally in patients unable to undergo other non-invasive imaging exams, i.e., magnetic resonance imaging (MRI), due to the presence of pacemakers/defibrillators MRI-unsafe or conditional or due to the artifacts generated by the metallic devices [84]. CCT can also be complementary to echocardiography and MRI, permitting an accurate assessment of coronary arteries (CA), and providing an accurate anatomical and functional assessment of cardiac chambers, along with the tissue characterization of the myocardium [85,86]. However, it is an advance cardiovascular image technique that uses ionizing radiations [84].

### 4.1. Protocols

In this setting, the myocardial structure and function evaluation can be as much as comprehensive utilizing the retrospective ECG-gated CCT imaging, where images are obtained through a sum of cardiac cycles, then retrospectively analyzed. This method provides information derived from the entire heart cycle (systole and diastole), permitting the examination of the global cardiac function at the expense of longer acquisitions and higher patient radiation exposure. To overcome the latter limitations, prospective CCT imaging techniques have been developed and implemented; in particular, the prospective CCT imaging in which the X-ray tube is activated only during the portions of the required/requested cardiac cycle (i.e., for CA imaging, typically in mid-diastole or end-systole), thus reducing radiation [87].

Regarding the CCT scanner’s utilization, CT technology has developed 16 cm wide detectors, dual-source, and dual-energy CCT scanners that allow the imaging acquisition of the entire cardiac volume in one heartbeat. In particular, dual-source CT is characterized by a reduction in gantry rotation time, permitting an increased temporal resolution that improves the end-systolic/end-diastolic phase identification [85,86]. This is important when examining pediatric on non-compliant patients [84].

The application of dual-energy CCT or delayed-phase cardiac CT allows the identification of myocardial scarring or fibrofatty replacement, likewise with delayed enhancement MRI [86].

Dual-energy CCT utilizes the distinctive absorption that human tissues and iodine-based contrast agents have when penetrated with different X-ray energy levels; these facilitate the iodine mapping distribution within the myocardium [88]. Similarly, delayed-phase CT scans, performed after a variable period of 5–15 min following coronary CT angiography, with the additional administration of contrast agent, are able to assess the presence of persistent contrast due to the slow washout of iodinated contrast from regions of replacement fibrosis or scarring [85]. Iodine is not the only element selectively identifiable by CCT. If the material density can be calculated by means of Hounsfield units, it can be determined if the tissue is composed of the myocardium or fluids of fat. This offers a complementary tool for myocardial characterization along with echocardiography or MRI, the gold standard [86,87].

### 4.2. Role of CT in Paediatric Cardiomyopathies

Intramyocardial fat is not always pathological, but it has been estimated that it frequently occurs in adults with normal hearts at a CCT. The latter increases with age and about 85% patients without cardiac disease have right ventricular (RV) fat in a postmortem examination [86,89,90].

**Figure 3:**
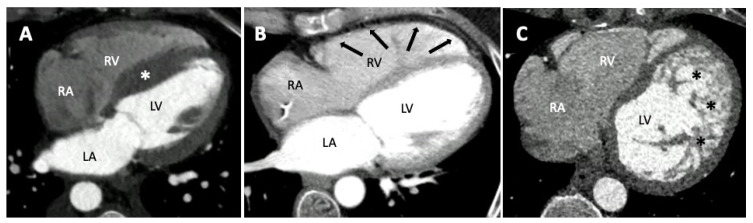
Cardiovascular computed tomography imaging presenting three patients with different cardiomyopathy. Panel (**A**): Hypertrophic Cardiomyopathy (HCM). The white asterisk indicates the hypertrophic septum. Panel (**B**): Arrhythmogenic cardiomyopathy right. The black arrows indicate the fibrofatty deposition of the right ventricle in the dual-energy CCT. Panel (**C**): Left ventricular non-compaction. The black asterisks indicate the prominent trabeculae of the left ventricle. Right ventricle (RV); left ventricle (LV); right atrium (RA); left atrium (LA).

Despite these premises, myocardial fibrofatty replacement is the most well recognized feature of arrhythmogenic cardiomyopathy (ACM). To date, the diagnostic criteria for ACM do not consider CCT, but RV dilatation with scalloping of the free wall, drastically thinned due to the fibrofatty deposition, or fat accumulation in prominent trabeculae and the moderator band, all appear to be suggestive for ACM in CCT (Figure 3) [91]. Additionally, left ventricular (LV) involvement can also be assessed and has been described most often as wedge-shaped myocardial defects in the free wall. Depending on the acquisition technique, it is possible to also see regional wall motion abnormalities and to assess ventricular function, often depressed in ACM [86,87,92].

As explained, most CCTs are performed in mid-diastole, with the exception of CTs focused on CA, that could also be in end-systole, where chambers quantification and wall thickness can be precisely measured and dilated cardiomyopathies (DCM), or hypertrophic cardiomyopathies (HCM) can be easily identified (Figure 3) [86,87,93]. LV non-compaction (LVNC) is easily identifiable in cardiac CT when high-resolution images are acquired, facilitating a better recognition of the areas in which the trabeculae are more prominent and distributed (Figure 3) [85,94].

### 4.3. Future Perspectives

The development and implementation of CCT is rapidly improving, remarkably by means of artificial intelligence but, at the moment, the application of CCT to cardiomyopathies can be achieved with basic CT techniques [86,87]. Therefore, CCT should be considered in clinical practice for the analysis of biventricular volume and function, and myocardial characterization, adjuvating MRI, or when the latter is contraindicated and echocardiographic exams are inconclusive.

Finally, CCT is gaining increasing attention in the context of cardiac transplants. Although its diagnostic role in preparation for transplantation is still limited, with a main role played by cardiac catheterization, CCT allows the performing of serial follow-up evaluations, aiming at diagnosing a possible onset of coronary graft disease after the transplantation [95].

## 5. Role of Nuclear Medicine Imaging in Pediatric Cardiomyopathies

Nuclear medicine imaging may be useful in the evaluation of cardiomyopathies in pediatric patients, offering important information regarding cardiac pump function and myocardial function and representing a valuable complement to other imaging techniques used for these purposes (such as echocardiography or cardiac magnetic resonance imaging) [96].

### 5.1. Protocols

For the evaluation of cardiac pump function, ECG-gated single photon emission computed tomography (SPECT), with 99mTc labelled radio compounds (sestamibi or tetrofosmin) or 201Tl, provides important information regarding left ventricular (LV) function, such as LV systolic and diastolic volumes and LV ejection fraction [97]. Considering the physiologic high heart rate often observed in pediatric patients, an acquisition of 10–12 gating intervals is recommended to keep an acceptable temporal resolution [98]. In this context, radionuclide ventriculography may represent a valid alternative for the evaluation of LV ejection fraction, LV or right ventricle (RV) dilatation, and kinesis [99].

### 5.2. Nuclear Imaging Application in Pediatric Cardiomyopathies

**Figure 4:**
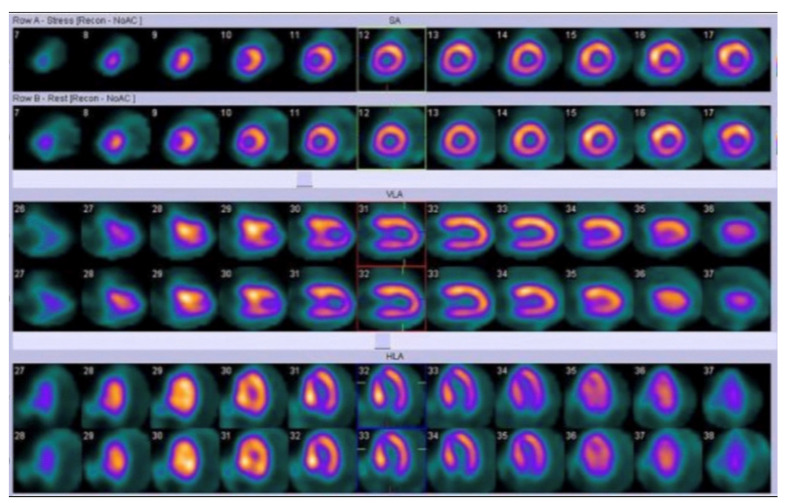
Myocardial perfusion imaging with Single Photon Emission Computed Tomography (SPECT) in a pediatric patient: both stress and rest images (axial images in first and second lines, vertical long axis images in third and fourth lines, horizontal long axis images in fifth and sixth lines) show left ventricular posterior wall and septal hypertrophy [98].

Significant aspects of myocardial function in pediatric cardiomyopathies, such as perfusion, viability, infections/inflammations, and cardiac innervation may be investigated in nuclear medicine. SPECT with 99mTc labelled radio compounds or 201Tl allows perfusion imaging, using physical stressors (for example cycle ergometer) or pharmacological stressors (such as dipyridamole or adenosine) [98]. In pediatric nuclear cardiology, pharmacological stressors require less compliance from patients, and they are often preferred in clinical practice [98]. It is important to underline that myocardial perfusion is a crucial aspect in evaluating children with cardiomyopathy (especially with hypertrophic cardiomyopathy); in fact, myocardial ischemia is strongly correlated with unfavorable outcomes in these patients, as shown in the study by Ziolkowska et al. in which 91 children with hypertrophic cardiomyopathy underwent 99mTc-sestamibi SPECT evaluation (Figure 4). The authors found that myocardial ischemia was a predictor of risk of death and adverse clinical events in pediatric patients [100]. In this regard, an interesting perfusion defect on myocardial scintigraphy was observed in patients with cardiomyopathy due to Duchenne muscular dystrophy and Becker muscular dystrophy. In fact, these patients often present myocardial perfusion reduction in the inferior wall of left ventricle, due to the wall stress in this region (dystrophin-deficient myocardium is typically vulnerable to pressure overload in comparison to normal myocardium) [101]. In the context of myocardial perfusion assessment in children with cardiomyopathy, positron emission tomography (PET), with radiopharmaceuticals such as 13N-ammonia, may allow a reliable quantification of coronary flow reserve; this parameter is often reduced in pediatric patients with hypertrophic cardiomyopathy, as highlighted in a previous study by Tadamura et al. The authors, evaluating six pediatric patients with hypertrophic cardiomyopathy, in comparison with six adults presenting the same condition and six healthy controls, found significant regional differences of coronary flow reserve (measured with 13N-ammonia PET) in pediatric patients (with an absolute reduction in myocardial blood flow in four of these six patients) [102]. A strong predictor of impaired coronary flow reserve, assessed with 13N-ammonia PET, is represented by maximum wall thickness, as demonstrated by Bravo et al. in a study performed in 33 patients with symptomatic hypertrophic cardiomyopathy [103].

In patients with cardiomyopathies, PET with 18F-fluorodeoxyglucose (18F-FDG, a radiopharmaceutical that acts as an analog of glucose) may represent a useful imaging technique for the assessment of myocardial vitality, providing metabolic information with high spatial resolution: the uptake of this radiopharmaceuticals by cardiomyocytes indicates metabolic activity and reflects cellular viability [104]. Furthermore, 18F-FDG PET is an interesting tool for the evaluation of infections/inflammations as well, due to the ability of cells involved in infections/inflammations to express high levels of glucose transporters (GLUT), especially neutrophils and monocyties/macrophages (in particular, these cells may present a high expression of GLUT1 and GLUT3) [105]. In clinical practice, 18F-FDG PET is often used for the evaluation of inflammatory and infectious diseases such as sarcoidosis, vasculitis and/or in the assessment of prostheses infection [100]. In the context of pediatric cardiomyopathies, 18F-FDG PET may allow a valid assessment of infection of left ventricular assist devices used in pediatric patients with end-stage heart failure, as shown in two interesting cases described by Absi et al. [106]. 18F-FDG PET may be also an interesting imaging technique for the detection of inflammatory areas in cardiac sarcoidosis, even in patients with preserved LV ejection fraction, as highlighted by Porcari et al.; furthermore, 18F-FDG PET may be used in the therapy-monitoring of these patients [107].

The use of radiopharmaceuticals analogues of catecholamines allows a cardiac innervation imaging: scintigraphy with 123I-metaiodobenzylguanidine (123I-MIBG) or PET with 11C-meta-hydroxyephedrine (11C-mHED), assessing pre-synaptic neuronal function, are useful imaging techniques for the evaluation of heart failure in children with cardiomyopathies, such as dilated cardiomyopathy [91,103,107]. In this context, Karasawa et al. demonstrated the role of myocardial scintigraphy with 123I-MIBG as a predictor of mortality and therapeutic outcomes in 33 pediatric patients with heart failure (including 8 children with cardiomyopathy) [108]. Moreover, an altered tracer uptake of 123I-MIBG is associated with a higher risk of ventricular tachyarrhythmias, especially in patients with arrhythmogenic right ventricular cardiomyopathy [99].

### 5.3. Limitations and Technical Considerations

Concerning the injected dose in pediatric patients, the main common limitation is related to the ionizing radiation exposure induced by nuclear medicine imaging. Therefore, the minimization of radiation exposure is mandatory in these patients: the ALARA (“as low as reasonably achievable”) principles should be followed, performing a dose reduction with algorithms as well (for example the Dosage Card of European Association of Nuclear Medicine) [98,108]. Nuclear medicine physicians should be also aware of the possible overestimation of parameters deriving from Gated SPECT mentioned before, often higher in small hearts [98]. Moreover, concerning other technical aspects, attenuation correction of SPECT images is performed with algorithms using computed tomography or iterative reconstruction techniques; attenuation correction with computed tomography is not recommended in children, in order to avoid additional radiation exposure [109,110,111,112].

## 6. Conclusions

**Figure 5:**
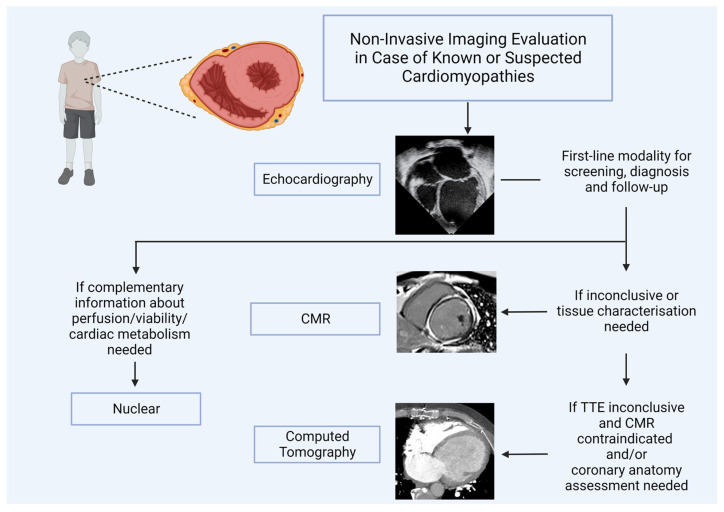
Cardiovascular imaging algorithm in the pediatric cardiomyopathy field.

Although echocardiography still represents the first-line imaging modality for evaluating children with known or suspected cardiomyopathies, the technological advances made in CMR, CT, and nuclear fields have led to the increasing use of these modalities in the pediatric age group. CMR should be used after echocardiography when not enough information is detected, and tissue characterization is required. In case it is contraindicated, CT could be a valuable alternative, whereas nuclear should be added if complementary information about perfusion, viability, and metabolism are necessary (Figure 5). A more appropriate imaging approach should be tailored to the diagnostic question, local availability, and expertise and should follow the ALARA principle when appropriate (Table 1).

Clinical trials or large-scale clinical studies addressing the unique characteristics of various pediatric cardiomyopathies, and their corresponding phenocopies detected through different imaging techniques, are currently lacking. The utilization of artificial intelligence holds promise in facilitating these studies by effectively handling extensive data sets akin to the human brain and identifying meaningful correlations within the data using machine learning techniques.

## Figures and Tables

**Table 1:** Advantages and disadvantages of cardiovascular imaging modalities in pediatric cardiomyopathy based on the previous sections.

	Advantages	Limitations
**Echocardiography**	Low cost Widely available Radiation free Excellent temporal resolution (evaluation of dynamic gradients or degree of associated valvular abnormalities) Diastolic function assessment Increased accuracy when using advanced echocardiographic techniques (strain, 3D-Echo)	High operator dependence Limited image quality in patients with poor acoustic windows Limited ability to discriminate between phenocopies
**Cardiovascular Magnetic Resonance**	Radiation free Gold standard for volumetric assessment (particularly for the right ventricle) Tissue characterization and fibrosis quantification	Longer acquisition time and cooperation required for breath-holding sequences Not widely available Higher cost compared to echocardiography Limitations in patients with non-conditional devices Potential risk of nephrogenic systemic fibrosis in patients with eGFR < 30 mL/min/1.73 m^2^ when using GBCAs (but less than 0.07% when using group II GBCAs)
**Nuclear Imaging**	Good spatial resolution Assessment of cardiac metabolism/inflammation Assessment of cardiac viability/perfusion	Exposure to radiation Management of radioactive tracers Possible overestimation of parameters deriving from gated SPECT
**Computed Tomography**	High spatial resolution Complementary assessment of coronary anatomy Assessment of volumes and function when other modalities are unconclusive or contraindicated	Exposure to ionizing radiation Potential risk of CIN with the use of iodinated contrast agent

## Data Availability

Not applicable.

## Abbreviations

ACM/ARVC	Arrhythmogenic Cardiomyopathy
bSSFP	Balanced Steady-State Free Precession
DCM	Dilated Cardiomyopathy
DSP	Desmoplakin
CA	Coronary Artery
CAD	Coronary Artery Disease
CT	Computed Tomography
CCT	Cardiovascular Computed Tomography
CMR	Cardiovascular Magnetic Resonance
ECV	Extracellular Volume
EGE	Early Gadolinium Enhancement
GBCA	Gadolinium-Based Contrast Agent
GLS	Global Longitudinal Strain
HCM	Hypertrophic Cardiomyopathy
HF	Heart Failure
LA	Left Atrium
LV	Left Ventricle
LVEDD	Left Ventricle End-Diastolic Diameter
LVEFLVESD	Left Ventricle Ejection FractionLeft Ventricle End-Systolic diameter
LVH	Left Ventricle Hypertrophy
LVM	Left Ventricle Mass
LVNC	Left Ventricular Non-Compaction Cardiomyopathy
LVOTO	Left Ventricular Outflow Tract Obstruction
NSF	Nephrogenic Systemic Fibrosis
RV	Right Ventricle
SAX	Short Axis
STE	Speckle Tracking Echocardiography
STIR	Short-TI Triple-Inversion Recovery Prepared Fast Spin Echo Sequences
TDI	Tissue Doppler Imaging
TR	Tricuspid Regurgitation
TTE	Transthoracic Echocardiography
2D	Two-Dimensional
3D	Three-Dimensional
